# 1-Quasiconformal Mappings and CR Mappings on Goursat Groups

**DOI:** 10.1155/2014/930571

**Published:** 2014-04-24

**Authors:** Qing Yan Wu, Zun Wei Fu

**Affiliations:** Department of Mathematics, Linyi University, Linyi, Shandong 276005, China

## Abstract

We show that 1-quasiconformal mappings on Goursat groups are CR or anti-CR mappings. This can reduce the determination of 1-quasiconformal mappings to the determination of CR automorphisms of CR manifolds, which is a fundamental problem in the theory of several complex variables.

## 1. Introduction

The well-known Liouville theorem tells us that 1-quasiconformal mappings on *R* ^*n*^ are elements of group SO(*n* + 1,1). Quasiconformal mappings on the Heisenberg group were introduced by Mostow [1] in his study of rigidity theorem. Since then, quasiconformal mappings on nilpotent Lie group and on more general Carnot-Carathéodory spaces had drawn more and more concern. Korányi and Reimann [2] and Korányi and Reimann [3] showed that *C* ^4^ orientation preserving 1-quasiconformal mappings between domains of Heisenberg group *H* ^*n*^ are CR or anti-CR mappings and are obtained by the restriction of SU(*n* + 1,1) group actions (see also [4]). Cowling et al. [5] extended Liouville theorem to conformal mappings on *G*/*P*, where *G* is a semisimple Lie group and *P* is a minimal parabolic subgroup of *G*. Ottazzi and Warhurst [6, 7] obtained a Liouville type theorem for all Carnot groups which sated that 1-quasiconformal maps form finite dimensional Lie groups. Xie [8] investigated 1-quasiconformal mappings on Carnot groups with reducible first layer equipped with left-invariant sub-Riemannian Carnot metrics and showed that each 1-quasiconformal map defined on a domain of such groups is the restriction of the composition of a standard dilation, a left translation, and an isometric graded isomorphism. In [9], we showed the CR property of 1-quasiconformal mappings on the Engel group and obtained the identity component of the group of 1-quasiconformal mappings. For other deformations of CR mappings between the nilpotent Lie group of step two and their Beltrami equations, one can refer to [10]. In this paper, we will investigate the 1-quasiconformal mappings on Goursat groups, which are stratified nilpotent Lie groups including Engel groups and a large class of high step Carnot groups.

A* Goursat group* is a nilpotent group endowed with a Goursat distribution *D*. A distribution *D* of corank *s* ≥ 2 on a manifold *M* is* Goursat* if the subsheaves *D* ^*i*^ of the tangent bundle defined inductively by *D* ^*i*+1^ = [*D* ^*i*^, *D* ^*i*^] (*i* = 1,2,…, *s*; *D* ^1^ = *D*; [*D* ^*i*^, *D* ^*i*^] denotes the sheaf of vector fields generated by *D* ^*i*^ and the Lie brackets [*X*, *Y*], *X*, *Y* ∈ *D* ^*i*^) correspond to distributions; that is, they have constant rank, and this rank is rank⁡ *D* ^*i*+1^ = rank⁡ *D* ^*i*^ + 1, *i* = 1,2,…, *s* [11]. By a distribution of rank  *d* we understand any subbundle of linear dimension *d* in the tangent bundle *TM*; its corank is dim⁡ *M* − *d*.

Our model of Goursat groups is *G*_*n*_ in [12], which takes *R* ^*n*+1^ as the underlying space with the group multiplication

(1) (x0,x1,…,xn)(x0′,x1′,…,xn′)  =(x0+x0′,x1+x1′,x2+x2′     +x0x1′,…,xn+xn′+x0xn−1′     +12x02xn−2′+⋯+1(n−1)!x0n−1x1′).

It is easy to see that its Lie algebra of left-invariant vector fields is given by

(2) X0=∂∂x0,Xj=∂∂xj+x0∂∂xj+1+⋯+x0n−j(n−j)!∂∂xn, j=1,…,n.

Then *X*_0_, *X*_1_,…, *X*_*n*_ span the tangent space everywhere and the only nontrivial commutation relations among these vector fields are

(3) $$\begin{array}{c} \left[X_{0},X_{j}\right]=X_{j+1},\;j=1,\ldots ,n-1. \end{array}$$

We use *g* to denote the Lie algebra and it splits into the direct sum

(4) $$\begin{array}{c} g=V_{1}⊕V_{2}⊕\cdots ⊕V_{n}, \end{array}$$

where the vector space *V*_1_ is spanned by the fields *X*_0_, *X*_1_ and the space *V*_*j*_, *j* = 2,…, *n*, is spanned by *X*_*j*_, respectively. It is clear to see that when *n* = 3, *G*_*n*_ is Engel group.

Let *d*_*c*_(·, ·) be the Carnot-Carathéodory distance on the Goursat group. A homeomorphism *f* : *Ω* → *Ω*′ between domains on the Goursat group *G* is* quasiconformal* if

(5) H(p)=limsup⁡r→0 H(p,r)=limsup⁡r→0max⁡dc(p,q)=r⁡dc(f(p),f(q))min⁡dc(p,q)=r⁡dc(f(p),f(q)), p∈Ω,

is uniformly bounded. If in addition ||*H*||_*∞*_ = sup⁡_*p*∈*Ω*_⁡*H*(*p*) ≤ *K* the homeomorphism *f* is *K-quasiconformal*.

On *R* ^2^, a 1-quasiconformal mapping is holomorphic or antiholomorphic. If we identify the Heisenberg group with a quadratic hypersurface in *C* ^*n*^, 1-quasiconformal mapping on the quadratic hypersurface is the restriction of a holomorphic or antiholomorphic mapping on *C* ^*n*^ [3]. This can be viewed as the higher dimensional case of the holomorphicity of 1-quasiconformal mapping. This phenomenon happens for Goursat group.

A* CR manifold* is a smooth manifold *M* equipped with a distribution *H* ⊂ *TM* of even rank 2*n* and a complex structure as endomorphisms *J*_*p*_ : *H*_*p*_ → *H*_*p*_ with *J*_*p*_^2^ = −id. Denote

(6) $$\begin{array}{c} T_{1,0}M=\ker\left(J_{p}-\sqrt{-1}\;\text{id}\right),\\ T_{0,1}M=\ker\left(J_{p}+\sqrt{-1}\;\text{id}\right). \end{array}$$

A CR manifold is supposed to satisfy the following integrability condition: [*Z*_1_, *Z*_2_] ∈ *C* ^*∞*^(*T*_1,0_ *M*), if *Z*_1_, *Z*_2_ ∈ *C* ^*∞*^(*T*_1,0_ *M*). The number *n* is called* CR dimension of M* and *k* = dim⁡ *M* − 2*n* is called the* codimension of M*. A smooth mapping *f* : *M*_1_ → *M*_2_ is called* CR* if

(7) $$\begin{array}{c} f_{*}T_{1,0}M_{1}\subset T_{1,0}M_{2}. \end{array}$$

CR manifolds appear naturally as embedded real submanifolds of complex manifolds. The distribution *H* is then defined as *H* = *TM*∩*i* *TM* and *J* is the restriction of the complex structure in the ambient complex manifold to *H*. We recall that, for a manifold *M* = {*ρ*_1_ = 0, *ρ*_2_ = 0,…, *ρ*_*k*_ = 0} with $\bar{\partial }\rho_{1}\wedge \bar{\partial }\rho_{2}\wedge \cdots \wedge \bar{\partial }\rho_{k}\neq 0$, the induced CR structure on *M* is given by *T*_1,0_ *M* = *T*_1,0_ *C* ^*n*^∩ *C* *TM* =  {*Z* ∈ *T*_1,0_ *C* ^*n*^ | *Zρ*_1_ = 0, *Zρ*_2_ = 0,…, *Zρ*_*k*_ = 0}, and dim⁡*T*_1,0_ *M* = *n* − *k*.

The Goursat group *G*_*n*_ can be realized as the real submanifold of *C* ^*n*^:

(8) Sn={(z,w2,…,wn)∈Cn ∣ Im⁡wk=−12kk!(zk+z¯k)−12k∑j=1k−11j!(k−j)!zjz¯k−j,  k=2,…,n},

where *z* = *x* + *iy*, *w*_*j*_ = *u*_*j*_ + *iv*_*j*_, *j* = 2,…, *n*, by the diffeomorphism *T* : *S*_*n*_ → *G*_*n*_ defined by

(9) T(z,w2,…,wn)=(z,Rew2,…,Rewn).

It is easy to check that

(10) Z=∂∂z−iRez∂∂w2−⋯−i(n−1)!(Rez)n−1∂∂wn,

where ∂/∂*z* = (1/2)((∂/∂*x*) − *i*(∂/∂*y*)), ∂/∂*w*_*j*_ = (1/2)((∂/∂*u*_*j*_) − *i*(∂/∂*v*_*j*_)), *j* = 2,…, *n*, is the complex tangent vector of *S*_*n*_, and we can define the standard CR structure on *S*_*n*_ by *T*_1,0_ *S*_*n*_ = *CZ* and dim⁡*T*_1,0_ *S*_*n*_ = 1. We can also define quasiconformal mappings on *S*_*n*_ by (5) with respect to its Carnot-Carathéodory distance defined in Section 2. There is one to one corresponding between 1-quasiconformal mappings on *G*_*n*_ and *S*_*n*_ via the diffeomorphism *T*. Therefore, if we want to study 1-quasiconformal mappings on *G*_*n*_, we can just study the 1-quasiconformal mappings on *S*_*n*_. For further details see Section 2.

Denote

(11) $$\begin{array}{c} P=\left\{\left(x_{0},x_{1},\ldots ,x_{n}\right)\in G_{n}\;|\;x_{2}=x_{3}=\cdots =x_{n}=0\right\}. \end{array}$$

We show that 1-quasiconformal mappings between domains on Goursat group *G*_*n*_ whose Pansu differentials preserve *P* are CR or anti-CR.

**Theorem 1**

Let *Ω* be a domain in *S*_*n*_. Suppose that *f* = (*f*_1_,…, *f*_*n*_) : *Ω* → *f*(*Ω*) is a 1-quasiconformal mapping with $D\tilde{f}(p)$ preserving *P*; then *f* is CR or anti-CR; that is, either

(12) $$\begin{array}{c} \bar{Z}f_{k}=0,\;k=1,2,\ldots ,n, \end{array}$$

or

(13) $$\begin{array}{c} Zf_{k}=0,\;k=1,2,\ldots ,n, \end{array}$$

where $\tilde{f}=T\circ f\circ T^{-1}$ and *p* is some *P*-differential point.

**Remark 2**

By this theorem the determination of 1-quasiconformal mappings is reduced to the determination of CR automorphisms of a submanifold in *C* ^*n*^. According to the extension theorem (Theorem 1.4 in [13]), locally, the smooth CR automorphisms of *S*_*n*_ are holomorphic automorphisms of *S*_*n*_, which is also an interesting problem.

**Remark 3**

In the cases of Heisenberg group and Engel group, the condition of $D\tilde{f}(p)$ preserving *P* in Theorem 1 can be omitted. However, by a similar method as in those cases, we cannot remove it, since it is inconvenient to use the expressions of Baker-Campbell-Hausdorff formula for groups of higher step.

## 2. Preliminaries

There is a natural homogeneous norm ||·|| on *G*_*n*_, given by

(14) $$\begin{array}{c} {||\left(x_{0},x_{1},\ldots ,x_{n}\right)||}^{2n!}={\left(x_{0}^{2}+x_{1}^{2}\right)}^{n!}+\overset{n}{\underset{k=2}{\sum }}{\left|x_{k}\right|}^{2n!/k}, \end{array}$$

and an associated pseudometric *d*, given by

(15) $$\begin{array}{c} d\left(p,q\right)=||q^{-1}p||, \end{array}$$

for *p*, *q* ∈ *G*_*n*_. The metric is left-invariant and is related to the dilations by the formula

(16) $$\begin{array}{c} d\left(\delta_{c}p,\delta_{c}q\right)=cd\left(p,q\right), \end{array}$$

where

(17) $$\begin{array}{c} \delta_{c}\left(x_{0},x_{1},\ldots ,x_{n}\right)=\left(cx_{0},cx_{1},c^{2}x_{2},\ldots ,c^{n}x_{n}\right),\;c\in R^{+}, \end{array}$$

denotes the dilation on *G*_*n*_.

The* horizontal tangent space at p* ∈ *G*_*n*_ is a subspace *H*_*p*_ *G*_*n*_ of the tangent space *T*_*p*_ *G*_*n*_, and it is spanned by the vector fields *X*_0_(*p*) and *X*_1_(*p*). An absolutely continuous curve *γ* : [0,1] → *G*_*n*_ is* horizontal* if its tangent vectors $\dot{\gamma }(t)$, *t* ∈ [0,1], lie in the horizontal tangent space *H*_*γ*(*t*)_ *G*_*n*_. By Chow [14], any given two points *p*, *q* ∈ *G*_*n*_ can be connected by a horizontal curve.

On *H*_*p*_ *G*_*n*_, we fix a quadric form 〈·, ·〉_*G*_ with respect to which the vector fields *X*_0_(*p*), *X*_1_(*p*) are orthonormal at every point *p* ∈ *G*_*n*_ and satisfy

(18) |αX0(p)+βX1(p)|G2  =〈αX0(p)+βX1(p),αX0(p)+βX1(p)〉G  =α2+β2,

where *α*, *β* ∈ *R*. 〈·, ·〉_*G*_ is called* infinitesimal Carnot-Carathéodory (C-C) metric*. Then the* length l*(*γ*) of the curve *γ* is defined by

(19) $$\begin{array}{c} l\left(\gamma \right)=\overset{1}{\underset{0}{\int }}{\langle \dot{\gamma }\left(t\right),\dot{\gamma }\left(t\right)\rangle }_{G}^{1/2}dt. \end{array}$$

The* Carnot-Carathéodory distance (C-C distance)* *d*_*c*_(*p*, *q*) is the infimum of the lengths of all absolutely continuous horizontal curves connecting *p* and *q*:

(20) $$\begin{array}{c} d_{c}\left(p,q\right)=\underset{\gamma }{\inf}l\left(\gamma \right). \end{array}$$

According to [15], this metric is equivalent to the pseudometric; that is,

(21) $$\begin{array}{c} C_{1}d\left(x,y\right)\leq d_{c}\left(x,y\right)\leq C_{2}d\left(x,y\right),\;\forall x,y\in G_{n}, \end{array}$$

for suitable constants *C*_1_, *C*_2_.

For the manifold *S*_*n*_, the horizontal tangent space at *x* ∈ *S*_*n*_ is *H*_*x*_ *S*_*n*_ = span⁡_*R*_{*ReZ*(*x*), *Im*⁡*Z*(*x*)}. On *H*_*x*_ *S*_*n*_, we fix a quadric form 〈·, ·〉_*S*_ with respect to which 2*ReZ*(*x*), 2*Im*⁡*Z*(*x*) are orthonormal at every point *x* ∈ *S*_*n*_ and satisfy

(22) |aReZ(x)+bIm⁡Z(x)|S2  =〈aReZ(x)+bIm⁡Z(x),aReZ(x)+bIm⁡Z(x)〉S  =a2+b24,

where *a*, *b* ∈ *R*. The C-C distance on *S*_*n*_ can be defined as above.

By definition (8), *S*_*n*_ in *C* × *C* ^*n*−1^ corresponding to *G*_*n*_ is defined by the equation

(23) $$\begin{array}{c} \rho_{1}=\rho_{2}=\cdots =\rho_{n-1}=0, \end{array}$$

where

(24) ρ1(z,w2,…,wn)=w2−w¯22i+14Rez2+14|z|2,ρ2(z,w2,…,wn)=w3−w¯32i+18Rez2z¯+124Rez3,⋮ρn−1(z,w2,…,wn)=wn−w¯n2i+12n∑j=1n−11j!(n−j)!zjz¯n−j +12nn!(zn+z¯n).

It is direct to check that

(25) $$\begin{array}{c} \bar{\partial }\rho_{1}\wedge \bar{\partial }\rho_{2}\wedge \cdots \wedge \bar{\partial }\rho_{n-1}\neq 0. \end{array}$$

And the vector field *Z* defined by (10) satisfies

(26) $$\begin{array}{c} Z\rho_{j}=0,\;\;\;j=1,\ldots ,n-1. \end{array}$$

That is, *Z* is a complex tangent vector field of *S*_*n*_, and *S*_*n*_ is a CR manifold with the induced CR structure *T*_1,0_ *S*_*n*_ = *CZ*. We obtain the following relation between *S*_*n*_ and *G*_*n*_.

**Proposition 4**

*S*_*n*_ is CR diffeomorphic and isometric to *G*_*n*_ via *T* defined by (9); that is, *T* and *T* ^−1^ are CR and isometric.

**Proof**

We can denote the point in *G*_*n*_ as (*z*, *x*_2_, *x*_3_,…, *x*_*n*_) where *z* = *x*_0_ + *ix*_1_, and then the group multiplication of *G*_*n*_ can be written in the following form:

(27) (z,x2,…,xn)(z′,x2′,…,xn′) =(z+z′,x2+x2′+(Rez)Im⁡z′,…,xn    +xn′+(Rez)xn−1′+12(Rez)2xn−2′    +⋯+1(n−1)!(Rez)n−1Im⁡z′).

Then the complex horizontal space is spanned by $Z_{G},{\bar{Z}}_{G}$, where

(28) ZG=12(X0−iX1)=∂∂z−i2(Rez)∂∂x2−⋯−i2(n−1)!(Rez)n−1∂∂xn;

here ∂/∂*z* = (1/2)((∂/∂*x*_0_) − *i*(∂/∂*x*_1_)).

We claim that, under this diffeomorphism *T* defined by (9), the complex tangent vector fields $Z,\bar{Z}$ of *S*_*n*_ are mapped onto *Z*_*G*_ and ${\bar{Z}}_{G}$, respectively. In fact, for any *f* ∈ *C* ^*k*^(*G*_*n*_) and *q* = (*z*, *w*_2_,…, *w*_*n*_) ∈ *S*_*n*_,

(29) (T∗Z)f(q) =Z(f∘T)(q) =[∂∂z−iRez∂∂w2−⋯−i(n−1)!(Rez)n−1∂∂wn][f(z,z¯,w2+w¯22,…,wn+w¯n2)] =∂f∂z−iRez2∂f∂x2−⋯−i2(n−1)!(Rez)n−1∂f∂xn =ZGf(T(q)).

Therefore,

(30) $$\begin{array}{c} T_{*}Z=Z_{G}. \end{array}$$

Similarly

(31) $$\begin{array}{c} T_{*}\bar{Z}={\bar{Z}}_{G}. \end{array}$$

It is clear to see that *T* ^−1^ : *G*_*n*_ → *S*_*n*_ is

(32) $$\begin{array}{c} T^{-1}\left(z,x_{2},\ldots ,x_{n}\right)=\left(z,x_{2}+iv_{2},\ldots ,x_{n}+iv_{n}\right), \end{array}$$

where

(33) vk=−12kk!(zk+z¯k)−12k∑j=1k−11j!(k−j)!zjz¯k−j,k=2,…,n.

Similarly, we find

(34) $$\begin{array}{c} T_{*}^{-1}Z_{G}=Z,\;\;T_{*}^{-1}{\bar{Z}}_{G}=\bar{Z}. \end{array}$$

Hence, *T*, *T* ^−1^ are CR and by (30), (31), and (34) together with (18) and (22), we get |*T*_∗_ *X*|_*G*_ = |*X*|_*S*_ for any *X* ∈ *H*_*q*_ *S*_*n*_ and |*T*_∗_^−1^ *Y*|_*S*_ = |*Y*|_*G*_ for any *Y* ∈ *H*_*p*_ *G*_*n*_, so *T*, *T* ^−1^ preserve C-C distance. This proves the proposition.

## 3. *P*-Differential of 1-Quasiconformal Mappings on *G*_*n*_

The notion of differential of a map between domains in Carnot group is due to Pansu [16]. Let *τ*_*p*_(*h*) = *ph* denote left-translation on *G*_*n*_. The mapping *f* : *Ω* → *Ω*′ (*Ω*, *Ω*′ are domains in *G*_*n*_) is called* p-differentiable at p* ∈ *Ω* if, for *c* → 0, the mappings

(35) $$\begin{array}{c} \delta_{c}^{-1}\circ \tau_{f\left(p\right)}^{-1}\circ f\circ \tau_{p}\circ \delta_{c} \end{array}$$

converge locally uniformly to a homomorphism *Df*(*p*) of the group *G*_*n*_, which is called* Pansu differential (P-differential)*.

The Pansu differential *Df*(*p*) is a Carnot homomorphism. Denote by *Df*_∗_(*p*) the induced Lie algebra homomorphism of the group homomorphism *Df*(*p*). According to [17], *Df*_∗_(*p*) is grading-preserving. As a result of [16] or [18], quasiconformal mappings between domains in Carnot group are *P*-differentiable a.e. The result is valid on the group *G*_*n*_ as it is a Carnot group.

Define the bilinear form *B* on *g* by

(36) $$\begin{array}{c} \left[X_{j},X_{k}\right]=B\left(X_{j},X_{k}\right)X_{2},\;\;j,k=0,1. \end{array}$$

Then by [*X*_0_, *X*_1_] = *X*_2_, the corresponding matrix is

(37) $$\begin{array}{c} B=\left(\begin{array}{cc} 0 & 1\\ -1 & 0 \end{array}\right). \end{array}$$

Let *f* be a 1-quasiconformal mapping between domains in *G*_*n*_; since *Df*_∗_(*p*) is grading-preserving, then *Df*_∗_(*p*) maps *V*_*j*_  (*j* = 1,…, *n*) onto itself, respectively. In particular *V*_1_ = span⁡{*X*_0_, *X*_1_}, so for any *X* ∈ *V*_1_,

(38) $$\begin{array}{c} Df_{*}\left(p\right)X=SX, \end{array}$$

for some 2 × 2 matrix *S*. Since *V*_2_ = *RX*_2_, then

(39) $$\begin{array}{c} Df_{*}\left(p\right)X_{2}=\lambda X_{2} \end{array}$$

for some *λ*.

**Proposition 5**

The matrix *S* above can be written as the following form:

(40) $$\begin{array}{c} S=\left(\begin{array}{cc} c\cos\theta & c\sin\theta \\ -\frac{\lambda }{c}\sin\theta & \frac{\lambda }{c}\cos\theta \end{array}\right), \end{array}$$

for some *θ* ∈ [0,2*π*), where *c* = |*Df*_∗_(*p*)*X*|_*G*_ for any *X* ∈ *V*_1_ with |*X*|_*G*_ = 1, *λ* ^2^/*c* ^4^ = 1; *λ*, *θ*, *c* depend on *p* ∈ *G*_*n*_.

**Proof**

Since *Df*_∗_(*p*)*X* = *SX*, *X* ∈ *V*_1_, now suppose

(41) $$\begin{array}{c} S=\left(\begin{array}{cc} s_{11} & s_{12}\\ s_{21} & s_{22} \end{array}\right); \end{array}$$

then

(42) $$\begin{array}{c} Df_{*}\left(p\right)X_{k}=s_{1k}X_{0}+s_{2k}X_{1},\;k=0,1. \end{array}$$

Because *Df*_∗_(*p*) is Lie algebra homomorphism, that is,

(43) $$\begin{array}{c} \left[Df_{*}\left(p\right)X_{k},Df_{*}\left(p\right)X_{l}\right]=Df_{*}\left(p\right)\left[X_{k},X_{l}\right], \end{array}$$

together with *Df*_∗_(*p*)*X*_2_ = *λX*_2_, we find that

(44) $$\begin{array}{c} B\left(s_{1k}X_{0}+s_{2k}X_{1},s_{1l}X_{0}+s_{2l}X_{1}\right)=\lambda B\left(X_{k},X_{l}\right), \end{array}$$

namely,

(45) $$\begin{array}{c} S^{T}BS=\lambda B. \end{array}$$

By Lemma 3.3 in [19], for the 1-quasiconformal mapping *f*, we have

(46) $$\begin{array}{c} \frac{\max_{X\in V_{1},|X{|}_{G}=1}{\left|Df_{*}\left(p\right)X\right|}_{G}}{\min_{X\in V_{1},|X{|}_{G}=1}{\left|Df_{*}\left(p\right)X\right|}_{G}}=1, \end{array}$$

where |·|_*G*_ is defined by (18), that is,

(47) $$\begin{array}{c} \frac{{\max_{X\in V_{1},|X{|}_{G}=1}\left|SX\right|}_{G}}{{\min_{X\in V_{1},|X{|}_{G}=1}\left|SX\right|}_{G}}=1. \end{array}$$

Therefore, there exists *c* > 0, such that

(48) $$\begin{array}{c} {\left|SX\right|}_{G}=c, \end{array}$$

for any *X* ∈ *V*_1_, |*X*|_*G*_ = 1. Then (1/*c*)*S* is an orthogonal matrix. Together with (45), we have

(49) $$\begin{array}{c} BS=\frac{\lambda }{c^{2}}SB, \end{array}$$

that is,

(50) $$\begin{array}{c} \left(\begin{array}{cc} 0 & 1\\ -1 & 0 \end{array}\right)\left(\begin{array}{cc} s_{11} & s_{12}\\ s_{21} & s_{22} \end{array}\right)=\frac{\lambda }{c^{2}}\left(\begin{array}{cc} s_{11} & s_{12}\\ s_{21} & s_{22} \end{array}\right)\left(\begin{array}{cc} 0 & 1\\ -1 & 0 \end{array}\right). \end{array}$$

Thus

(51) s21=−λc2s12,s22=λc2s11,s11=λc2s22,s12=−λc2s21.

Therefore, *λ* ^2^/*c* ^4^ = 1. Note that ((1/*c*)*S*)^*T*^((1/*c*)*S*) = *I*, where *I* is the 2 × 2 unit matrix; hence

(52) s112+s122=c2,s212+s222=c2,s11s21+s12s22=0.

From (51), (52) and setting *s*_11_ = *c*cos⁡*θ*, *θ* ∈ [0,2*π*), we have (40). The result follows.

We get the following relation between *P*-differential and the usual derivatives. For the corresponding result on Heisenberg group see p.31 of [3]. In the following, the letter *C* will be used to denote various constants, and the various uses of the letter do not, however, denote the same constant.

**Proposition 6**

Suppose *f* is a quasiconformal mapping between domains in *G*_*n*_ with *f*(0) = 0, and if *f* is *P*-differentiable at 0 and *Df*(0) preserves the plan *P* in (11), then *f* is differentiable in the usual sense at 0 along the horizontal directions and

(53) $$\begin{array}{c} Df_{*}\left(0\right)\partial_{x_{j}}=f_{*0}\partial_{x_{j}},\;j=0,1, \end{array}$$

where *f*_∗0_∂_*x*_*j*__ = ∑_*k*=0_^*n*^(∂*f*_*k*_/∂*x*_*j*_)(0)∂_*x*_*k*__ and *Df*_∗_(0) is the P-derivative of *f* at 0.

**Proof**

Since *f* is *P*-differentiable at 0, by definition, there exists 0 < *δ* < 1 such that, for *c* < *δ* and ||(*z*, *x*_2_,…, *x*_*n*_)|| ≤ 1,

(54) ||(Df(0)(z,x2,…,xn))−1  ×(δc−1∘τf(0)−1∘f∘τ0∘δc(z,x2,…,xn))||=||(Df(0)(z,x2,…,xn))−1  ×(δc−1∘f∘δc(z,x2,…,xn))||<ɛ.

Recall that the homomorphism *Df*(0) commutes with *δ*_*c*_; then for points (*z*/|*z*|, 0,…, 0) in particular, we have

(55) ||δc−1((Df(0)(cz|z|,0,…,0))−1f(cz|z|,0,…,0))|| =1c||(Df(0)(cz|z|,0,…,0))−1f(cz|z|,0,…,0)||<ɛ.

Therefore

(56) 1|z|||(Df(0)(z,0,…,0))−1f(z,0,…,0)||<ɛ,if  |z|<δ.

Now compare this with Euclidean distances. Since *Df*(0) preserves the plane  *P*, that is,

(57) $$\begin{array}{c} Df\left(0\right)\left(z,0,\ldots ,0\right)=\left(\tilde{z},0,\ldots ,0\right)=\left({\tilde{x}}_{0},{\tilde{x}}_{1},0,\ldots ,0\right) \end{array}$$

for some $\tilde{z}=({\tilde{x}}_{0},{\tilde{x}}_{1})\in R^{2}$, then

(58) (Df(0)(z,0,…,0))−1  =(x~0,x~1,0,…,0)−1  =(−x~0,−x~1,x~0x~1,−x~022x~1,…,(−1)nx~0n−1(n−1)!x~1).

Assume that (*x*_0_′, *x*_1_′, *x*_2_′,…, *x*_*n*_′) = *f*(*z*, 0,…, 0); we get

(59) ||(Df(0)(z,0,…,0))−1f(z,0,…,0)||2n! =||(−x~0,−x~1,x~0x~1,−x~022x~1,…,(−1)nx~0n−1(n−1)!x~1)   ×(x0′,x1′,x2′,…,xn′)||2n! =(|x0′−x~0|2+|x1′−x~1|2)n!+|x2′−x~0(x1′−x~1)|2n!/2  +|x3′−x~0x2′+(−x~0)22(x1′−x~1)|2n!/3+⋯  +|xn′−x~0xn−1′+(−x~0)22xn−2′+⋯    +(−x~0)n−1(n−1)!(x1′−x~1)|2n!/n <ε2n!|z|2n!.

It follows that

(60) |x0′−x~0|2+|x1′−x~1|2<ε2|z|2,|x2′−x~0(x1′−x~1)|<ε2|z|2,|x3′−x~0x2′+(−x~0)22(x1′−x~1)|<ε3|z|3,         ⋮|xn′−x~0xn−1′+(−x~0)22xn−2′+⋯ +(−x~0)n−1(n−1)!(x1′−x~1)|<εn|z|n.

Therefore,

(61) $$\begin{array}{c} \left|x_{0}^{\prime }-{\tilde{x}}_{0}\right|<ɛ\left|z\right|,\;\;\left|x_{1}^{\prime }-{\tilde{x}}_{1}\right|<ɛ\left|z\right|, \end{array}$$

(62) |x2′|<ɛ2|z|2+ɛ|x~0||z|,|x3′|<ɛ3|z|3+|x~0||x2′|+(−x~0)22ɛ|z|  ⋮|xn′|<ɛn|z|n+|x~0||xn−1′|+(−x~0)22|xn−2′|+⋯   +ɛ|(−x~0)n−1(n−1)!||z|.

From Lemma 3.3 in [19], *Df*(0) is Lipschitz; then by the equivalence of *d*_*c*_ and *d*, we have

(63) $$\begin{array}{c} ||Df\left(0\right)\left(z,0,\ldots ,0\right)||\leq C{\text{Lip}}_{f}\left(0\right)||\left(z,0,\ldots ,0\right)||, \end{array}$$

for some *C* > 0, namely,

(64) $$\begin{array}{c} \left|{\tilde{x}}_{0}\right|\leq ||Df\left(0\right)\left(z,0,\ldots ,0\right)||\leq C{\text{Lip}}_{f}\left(0\right)\left|z\right|=M_{1}\left|z\right|. \end{array}$$

Since *f* is continuous, then when |*z*| < *δ* < 1, we have

(65) $$\begin{array}{c} \left|\left(x_{0}^{\prime },x_{1}^{\prime },x_{2}^{\prime },\ldots ,x_{n}^{\prime }\right)\right|\leq \left|f\left(z,0,\ldots ,0\right)\right|<M_{2}, \end{array}$$

where *M*_2_ is a positive constant. Now substituting (64) and (65) into (62) and then by iteration, we obtain

(66) |x2′|<ɛ2|z|2+ɛM1|z|2<Cɛ|z|2,|x3′|<ɛ3|z|3+|x~0||x2′|+(−x~0)22ɛ|z|  <ɛ3|z|3+Cɛ|x~0||z|2+(−x~0)22ɛ|z|<Cɛ|z|3,   ⋮|xn′|<ɛn|z|n+|x~0||xn−1′|+(−x~0)22|xn−2′|+⋯   +ɛ|(−x~0)n−1(n−1)!||z|  <ɛ2|z|n+Cɛ|x~0||z|n−1+Cɛ(−x~0)22|z|n−2+⋯   +Cɛ|(−x~0)n−1(n−1)!||z|  <Cɛ|z|n.

Then by (61), for any |*z* | <*δ* < 1, we have

(67) 1|z||Df(0)(z,0,…,0)−f(z,0,…,0)| =1|z||(x~0,x~1,0,…,0)−(x0′,x1′,x2′,…,xn′)| =1|z|(|x0′−x~0|2+|x1′−x~1|2+∑k=2n|xk′|2)1/2 <C1|z|(ɛ2|z|2+∑k=2nɛ2|z|2k)1/2 <Cɛ.

If we let *z* = (*x*_0_, 0), then

(68) 1|x0||Df(0)(x0,0,…,0)−f(x0,0,…,0)| =|Df(0)(1,0,…,0)−f(x0,0,…,0)x0| <Cɛ,

namely,

(69) $$\begin{array}{c} Df\left(0\right)\left(1,0,\ldots ,0\right)=\underset{x_{0}\to 0}{\lim}\frac{f\left(x_{0},0,\ldots ,0\right)-f\left(0\right)}{x_{0}}=\frac{\partial f}{\partial x_{0}}\left(0\right). \end{array}$$

Therefore,

(70) $$\begin{array}{c} Df_{*}\left(0\right)\partial_{x_{0}}=\overset{n}{\underset{j=0}{\sum }}\frac{\partial f_{j}}{\partial x_{0}}\left(0\right)\partial_{x_{j}}=f_{*0}\partial_{x_{0}}. \end{array}$$

Similarly

(71) $$\begin{array}{c} Df_{*}\left(0\right)\partial_{x_{1}}=\overset{n}{\underset{j=0}{\sum }}\frac{\partial f_{j}}{\partial x_{1}}\left(0\right)\partial_{x_{j}}=f_{*0}\partial_{x_{1}}. \end{array}$$

The proposition is proved.

We need the following relation between *P*-differential and the usual tangential mapping. See Proposition 3.2 in [10] for the corresponding result on the Heisenberg group.

**Proposition 7**

Let *f* : *Ω*_1_ → *Ω*_2_ be a quasiconformal mapping between domains in the Goursat group *G*_*n*_. Suppose *f* is *P*-differentiable at *p* and *Df*(*p*) preserves the plan *P* in (11); then

(72) $$\begin{array}{c} f_{*}\left(p\right)X\left(p\right)={\left(\tau_{f(p)}\right)}_{*0}\left[Df_{*}\left(p\right)X\left(0\right)\right], \end{array}$$

for *X* ∈ *V*_1_, where *f*_∗_(*p*)*X*(*p*) = ∑_*k*=0_^*n*^ *Xf*_*k*_(*p*)∂_*x*_*k*__ and *τ*_*q*∗_ are the tangential mappings of *τ*_*q*_ for *q* ∈ *G*_*n*_.

**Proof**

Suppose *f* is *P*-differentiable at *p* ∈ *G*_*n*_; let *F* = *τ*_*f*(*p*)_^−1^∘*f*∘*τ*_*p*_; then *F* is also a quasiconformal mapping and *F*(0) = 0. Moreover, by Chain rule (Lemma 3.7 in [19]), *F* is *P*-differentiable at the origin 0, and

(73) $$\begin{array}{c} DF\left(0\right)=D\tau_{f\left(p\right)}^{-1}\left(f\left(p\right)\right)Df\left(p\right)D\tau_{p}\left(0\right). \end{array}$$

Since *Dτ*_*p*_ is the identity automorphism, then

(74) $$\begin{array}{c} DF\left(0\right)=Df\left(p\right). \end{array}$$

Therefore, *DF*(0) preserves the plan *P*.

By Proposition 6, we have

(75) $$\begin{array}{c} DF_{*}\left(0\right)\partial_{x_{j}}=F_{*0}\partial_{x_{j}},\;j=0,1. \end{array}$$

From (2) we can see that *X*_0_(0) = ∂_*x*_0__ and *X*_1_(0) = ∂_*x*_1__. Therefore,

(76) $$\begin{array}{c} DF_{*}\left(0\right)X_{j}\left(0\right)=F_{*0}X_{j}\left(0\right),\;j=0,1. \end{array}$$

Consequently,

(77) f∗(p)Xj(p) =(τf(p)∘F∘τp−1)∗pXj(p) =(τf(p))∗0F∗0Xj(0) =(τf(p))∗0[DF∗(0)Xj(0)] =(τf(p))∗0[Df∗(p)Xj(0)],      j=0,1.

This completes the proof.

We need the following proposition due to Capogna ([19], Theorem 1.1).

**Proposition 8**

Suppose that *Ω*_1_ and *Ω*_2_ are open subsets of Carnot groups *G*_1_ and *G*_2_, and *f* : *Ω*_1_ → *Ω*_2_ is 1-quasiconformal. Then *f* is smooth.

**Proof of Theorem 1**

Let *f* be a 1-quasiconformal mapping satisfying the hypothesis in Theorem 1. By Proposition 4, *T*, *T* ^−1^ defined by (9) and (32) are CR and isometric; then $\tilde{f}=T\circ f\circ T^{-1}$ is a 1-quasiconformal mapping between domains in *G*_*n*_. Suppose *p* ∈ *G*_*n*_ is a *P*-differentiable point of $\tilde{f}$; then $D{\tilde{f}}_{*}(p)$ is grading-preserving; that is, $D{\tilde{f}}_{*}(p)X_{2}=\lambda X_{2}$ for some *λ*, and $D{\tilde{f}}_{*}X=SX,X\in V_{1}$. From Proposition 5, at *p* ∈ *G*_*n*_, *S* has form (40), and *λ* = ±*c* ^2^, where $c=|D{\tilde{f}}_{*}(p)X{|}_{G}$ for any *X* ∈ *V*_1_ with |*X*|_*G*_ = 1.

In the complexified subspace *CV*_1_, the corresponding complex basis is *Z*_*G*_, ${\bar{Z}}_{G}$, where *Z*_*G*_ is as (28). On the matrix level

(78) $$\begin{array}{c} S⟶S_{c}=\Theta S\Theta^{-1}, \end{array}$$

with $\Theta =\left(\begin{array}{cc} 1 & i\\ 1 & -i \end{array}\right)$. Then

(79) $$\begin{array}{c} S_{c}=\left(\begin{array}{cc} \frac{c^{2}+\lambda }{2c}e^{-i\theta } & \frac{c^{2}-\lambda }{2c}e^{i\theta }\\ \frac{c^{2}-\lambda }{2c}e^{-i\theta } & \frac{c^{2}+\lambda }{2c}e^{i\theta } \end{array}\right),\;\theta \in \left[0,2\pi \right). \end{array}$$

We claim that $D{\tilde{f}}_{*}(p)Z_{G}=S_{c}Z_{G}$, $D{\tilde{f}}_{*}(p){\bar{Z}}_{G}=S_{c}{\bar{Z}}_{G}$. In fact, by (28) and (40), we have

(80) Df~∗(p)ZG =Df~∗(p)[12(X0−iX1)] =12[cX0cos⁡θ−λcX1sinθ−i(cX0sinθ+λcX1cos⁡θ)] =12[ce−iθX0−iλce−iθX1] =12e−iθ[c(ZG+Z¯G)+λc(ZG−Z¯G)] =12e−iθ[(c+λc)ZG+(c−λc)Z¯G]=ScZG.

Similarly, $D{\tilde{f}}_{*}(p){\bar{Z}}_{G}=S_{c}{\bar{Z}}_{G}$.

From Proposition 8, $\tilde{f}$ is smooth, and therefore $f=T^{-1}\circ \tilde{f}\circ T$ is also smooth. We write the 1-quasiconformal mapping *f* in the form

(81) f(z,w2,…,wn)=(f1,f2,…,fn)(z,w2,…,wn)=(ω1,ω2,…,ωn)

with

(82) Im⁡fk=−12kk!(f1k+f¯1k)−12k∑j=1k−11j!(k−j)!f1jf¯1k−j,

where *k* = 2,3,…, *n*. Then

(83) $$\begin{array}{c} f_{*}Z=U+\bar{V}, \end{array}$$

where

(84) $$\begin{array}{c} U=\overset{n}{\underset{k=1}{\sum }}\left(Zf_{k}\right)\frac{\partial }{\partial \omega_{k}},\;\;\bar{V}=\overset{n}{\underset{k=1}{\sum }}\left(Z{\bar{f}}_{k}\right)\frac{\partial }{\partial {\bar{\omega }}_{k}}. \end{array}$$

In fact, for any *φ* ∈ *C* ^*k*^(*S*_*n*_),

(85) (f∗Z)φ=Z(φ∘f)=Z[φ(f1,f2,…,fn,f¯1,f¯2,…,f¯n)]=∑k=1n∂φ∂ωkZfk+∑k=1n∂φ∂ω¯kZf¯k=[∑k=1n(Zfk)∂∂ωk+∑k=1n(Zf¯k)∂∂ω¯k]φ.

Let *q* = *T* ^−1^(*p*) ∈ *S*_*n*_; by (30), (34), (72), and (80) together with the left-invariant property of *Z*_*G*_,

(86) f∗q(Z(q))=(T−1∘f~∘T)∗q(Z(q))=T∗−1∘f~∗∘T∗q(Z(q))=T∗−1∘f~∗p(ZG(p))=T∗−1∘τf~(p)∗0∘[Df~∗(p)ZG(0)]=T∗f~(p)−1∘τf~(p)∗0 ×(c2+λ2ce−iθZG(0)+c2−λ2ceiθZ¯G(0))=T∗f~(p)−1(c2+λ2ce−iθZG(f~(p))       +c2−λ2ceiθZ¯G(f~(p)))=c2+λ2ce−iθZ(f(q))+c2−λ2ceiθZ¯(f(q)).

Since *f* is *P*-differentiable a.e., we have

(87) $$\begin{array}{c} f_{*}Z=\frac{c^{2}+\lambda }{2c}e^{-i\theta }Z+\frac{c^{2}-\lambda }{2c}e^{i\theta }\bar{Z},\;\text{a}.\text{e}. \end{array}$$

Consequently, $U,\bar{V}$ in (83) have the following forms

(88) $$\begin{array}{c} U=\overset{n}{\underset{k=1}{\sum }}\left(Zf_{k}\right)\frac{\partial }{\partial \omega_{k}}=\frac{c^{2}+\lambda }{2c}e^{-i\theta }Z,\;\text{a}.\text{e}.,\\ \bar{V}=\overset{n}{\underset{k=1}{\sum }}\left(Z{\bar{f}}_{k}\right)\frac{\partial }{\partial {\bar{\omega }}_{k}}=\frac{c^{2}-\lambda }{2c}e^{i\theta }\bar{Z},\;\text{a}.\text{e}. \end{array}$$

Hence, if *λ* = *c* ^2^, then $\bar{Z}f_{k}=0$, *k* = 1,2,…, *n*; if *λ* = −*c* ^2^, then *Zf*_*k*_ = 0, *k* = 1,2,…, *n*. Since *Z* and *f* are smooth, Theorem 1 follows.

**Remark 9**

The CR automorphism of *G*_*n*_ (*n* > 3) is unknown now. It is also very interesting to determine it, since if this can be solved, we can get the 1-quasiconformal mappings on these groups.
